# Light‑Driven Propulsion of Graphene Aerogels in Microgravity

**DOI:** 10.1002/advs.75050

**Published:** 2026-03-31

**Authors:** Omnia Khattab, Rami Elkaffas, Basel Altawil, Omar Alsuwaidi, Afnan Almubashir, Claire Perfetti, Shanavas Shajahan, Marco Braibanti, Sean Swei, Carlo Saverio Iorio, Yarjan Abdul Samad

**Affiliations:** ^1^ Department of Aerospace Engineering Khalifa University of Science and Technology Abu Dhabi United Arab Emirates; ^2^ Khalifa University Space Technology and Innovation Lab Khalifa University Abu Dhabi United Arab Emirates; ^3^ Centre for Research and Engineering in Space Technologies Université libre de Bruxelles Brussels Belgium; ^4^ European Space Agency (ESA) ESTEC Noordwijk The Netherlands; ^5^ Cambridge Graphene Center University of Cambridge Cambridge UK; ^6^ Advanced Research and Innovation Center (ARIC) Khalifa University Abu Dhabi United Arab Emirates

**Keywords:** aerogel, graphene, microgravity, parabolic flight, weightlessness

## Abstract

Ultralight graphene aerogels can convert light into mechanical work, yet their performance under reduced gravity remains largely unquantified. We investigated light‐driven motion of macroscopic porous graphene networks during the 86th parabolic flight campaign of ESA, directly comparing their response in microgravity and at 1 g. In microgravity, optical excitation produced rapid translation: aerogels with a density of 0.01 g cm^−^^3^ traversed 50 mm within 0.05 s and reached peak velocities of 1.7 m s^−^^1^, with peak accelerations ≳10^2^ m s^−^^2^ and an initial thrust pulse of 0.6 mN occurring within 0.03 s. Under 1 g, motion was strongly suppressed: displacement peaked near ≈15 mm by ≈0.16 s, with a modest velocity maximum ≈0.06 m s^−^^1^ and a delayed thrust peak ≈11 µN at ≈0.08 s. Removing weight and normal‐force friction, therefore, markedly amplifies optically induced forces in these ultralow‐density networks. By directly quantifying distance, velocity, and transient thrust across gravitational regimes, our study establishes performance benchmarks for light‐driven propulsion using graphene aerogels in microgravity environments.

## Graphical abstract

Ultralight graphene aerogels convert laser light into thrust. Parabolic‐flight experiments directly compare their motion in microgravity and at 1 g, revealing order‐of‐magnitude enhancements in distance, velocity, and transient thrust when weight and friction are removed (∼50 times enhancement). The results benchmark light‐driven propulsion of porous graphene networks for future micro‐propulsion applications.

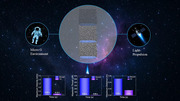

## 1 Introduction

Microgravity is defined as a state of near‐weightlessness, typically experienced during free‐fall conditions, such as in spacecraft orbiting Earth [1]. A parabolic flight is a ground‐based analog method that can provide microgravity, or weightlessness, similar to what is experienced during spaceflight, but for shorter time periods [2]. During a parabolic flight, an aircraft flies in repeated parabolic trajectories. Each arc consists of a steep climb followed by a sharp descent, creating short periods of free fall (0 g), which alternate with phases of increased gravitational force (high‐g pullouts) [3]. The aircraft typically achieves up to 20 seconds of microgravity per parabola, which is the window of the experimental testing [4]. For parabolic flights, the residual accelerations experienced by experiments are typically in the order of 10^−2^ to 10^−3^ [5].

Graphene is a 2D material composed of a single layer of carbon atoms arranged in a hexagonal honeycomb lattice structure. It is renowned for its unique properties. The thermal conductivity of single‐layer graphene with high structural perfection is extremely high, reaching up to 5000 W/mK [6]. However, this value decreases rapidly as the number of layers increases, approaching approximately 3000 W/mK. This remarkable performance is attributed to its high phonon group velocities and long mean free paths, which are crucial for efficient heat transport [7].  Graphene's Young's modulus can reach 1 TPa, and its tensile strength can reach 130 GPa [8]. Graphene aerogels are a type of 3D graphene network [9]. They are lightweight, porous materials that combine the unique properties of graphene with the structural benefits of aerogels [9]. Graphene aerogel densities can range from 0.00016 g/cm^3^ to 0.5 g/cm^3^ [10]. Graphene‐based aerogels exhibit good electrical conductivity (a few 10 s to 100 s of S/m) [11], adjustable porosity in a few to hundreds of µm range [12], and excellent mechanical performances; it has values of Young's modulus up to 20MPa [13].

In recent years, only a few studies have explored the intricate dynamics of light interactions with Graphene and Related Materials (GRMs). The interaction of light with graphene and its derivatives has revealed a wide spectrum of motion phenomena, ranging from levitation and rotation to bulk propulsion. One of the earliest demonstrations of optically controlled motion in graphene was achieved by Kane et al. in 2010 [14], who levitated graphene flakes in an electric quadrupole ion trap. By charging the flakes and injecting them into the trap, circularly polarized light was used to induce rotational motion. This system not only allowed for the study of fundamental angular momentum transfer in low‐pressure environments but also suggested potential for graphene manipulation through controlled optical torque. A different approach was proposed by Kobayashi and Abe [15], who demonstrated magnetic levitation of graphite flakes under strong magnetic fields. The motion of these flakes was modulated by photoirradiation, which altered their magnetic susceptibility. This light‐induced change enabled the optical control of translational and rotational dynamics, providing early evidence of photothermal modulation in layered carbon structures. At the macroscale, Zhang et al. [16] reported the direct light propulsion of bulk graphene materials, marking a significant leap in demonstrating Newtonian recoil driven by photon‐induced electron emission. Graphene's broadband light absorption and low density enabled efficient conversion of photonic energy into mechanical motion, highlighting its promise in solar sail propulsion and space transportation concepts. Further investigations into propulsion mechanisms were conducted by Wu et al. [17], who proposed that radiometric forces, arising from differential heating due to light absorption, might be responsible for the observed motion of graphene sponge samples. This mechanism operates independently of charge ejection and reflects the significance of thermally induced pressure differentials in rarefied gases. Building on this, Wang et al. [18] provided a more quantitative account of graphene sponge propulsion, attributing it primarily to the Knudsen force. This phenomenon arises from laser‐induced thermal nonequilibrium in a low‐pressure environment, resulting in a temperature gradient that generates a directional force. The method was shown to be highly efficient, stable, and scalable, offering an attractive path toward propellant‐free propulsion in microscale devices and optomechanical systems. Finally, in 2022, Xiao et al. [19] investigated graphene bubbles, where laser‐induced local heating and non‐uniform temperature distributions resulted in measurable motion. This nanoscale manipulation mechanism provides further evidence of light‐responsive behavior in graphene, reinforcing its relevance for optoelectronic and nanoactuation applications. Together, these studies establish a continuum of photonic interaction regimes in graphene‐based systems, from ion‐trap levitation to bulk and nanoscale propulsion. Each mechanism, whether governed by electromagnetic torque, thermal recoil, radiometric pressure, or Knudsen forces, offers insight into how light energy can be harnessed for mechanical actuation in graphene aerogels, thereby informing the current work.

The motivation of this work is to systematically examine the behavior of graphene aerogels subjected to optical propulsion under microgravity conditions. Although considerable progress has been made in understanding their response on the ground, the effects of gravity on these phenomena remain insufficiently characterized. By performing controlled experiments during parabolic flight and directly comparing the outcomes to those obtained under standard gravity on the ground, this study aims to identify distinct features of optically induced motion that arise in the microgravity environment. Such a comparative analysis is critical for advancing the fundamental understanding of graphene‐based materials and informing their potential application in future space technologies such as laser‐driven micro‐propulsion systems, attitude‐control actuators for small satellites, and ultralight graphene‐based solar sails for photon‐driven spacecraft [20].

## 2 Results and Discussion

### 2.1 Experimental Set‐Up and Parabolic Flight Configuration

**FIGURE 1:**
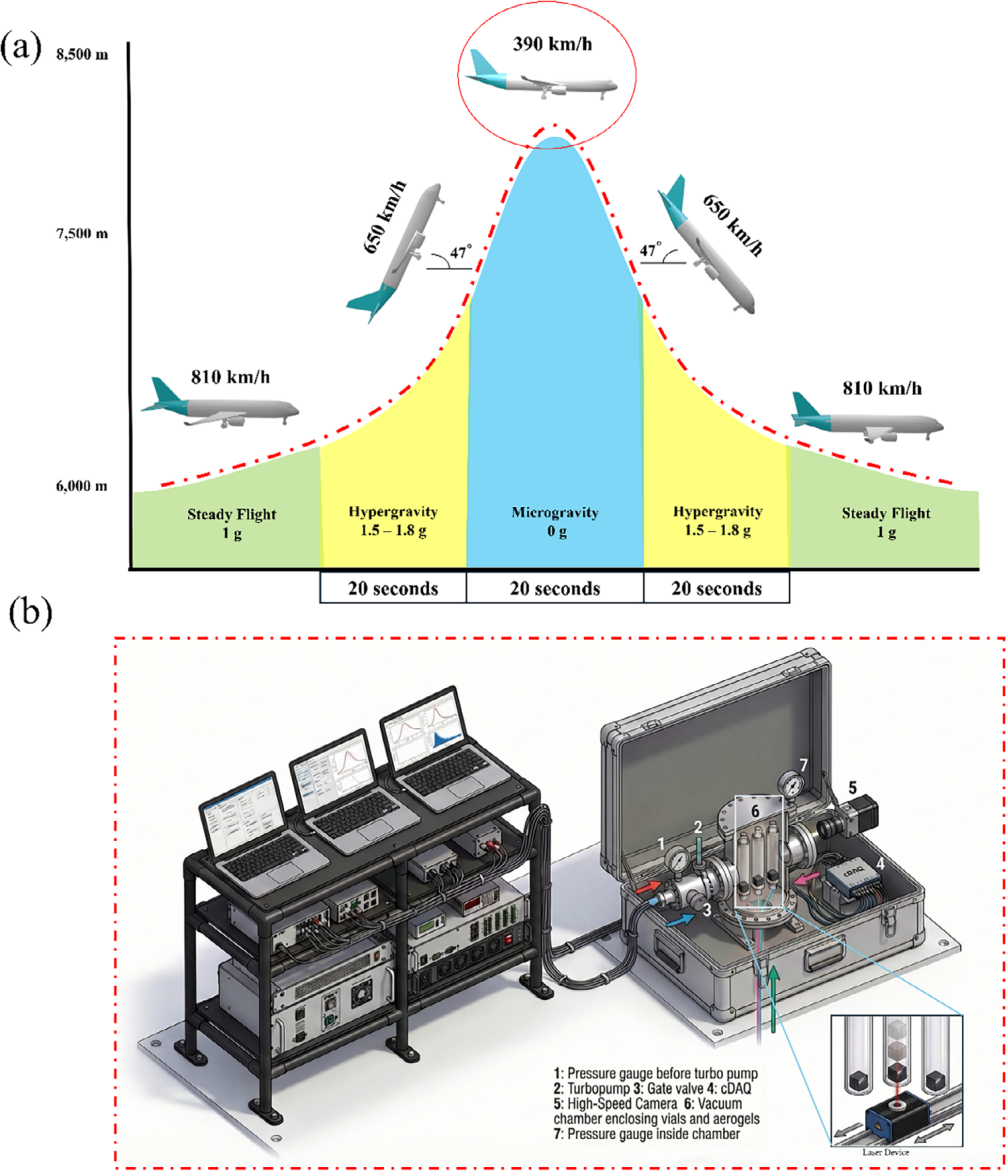
(a) Schematic representation of the parabolic flight. (b) Digital Images of the setup on the plane.

Parabolic flight is a controlled aerial maneuver employed to reproduce microgravity conditions (Figure 1a). The sequence begins with a steep ascent, typically reaching an angle of around 47° from steady horizontal flight at 1 g, resulting in an increased gravitational load of approximately 1.8 g. This is followed by the “injection” phase, during which engine thrust is reduced, and the aircraft transitions into a parabolic descent (free‐fall). Throughout this trajectory, the vertical load factor approaches zero, effectively creating a microgravity environment. Subsequently, the aircraft enters the “pull‐out” or recovery phase, descending at an angle of around 47°, during which the gravitational load again increases to around 1.8 g. The maneuver concludes with a return to level flight at 1 g. Each phase, hypergravity, microgravity, and recovery, typically lasts between 20 s and 25 s.

During the free‐falling phase of the parabola, all parts of the payload accelerate at the same rate as the local gravitational field, so the specific force (proper acceleration) measured by an onboard accelerometer approaches zero. In vector form, the non‐gravitational specific force f is

$$f=a_{aircraft}+a_{non-ideal}$$

where a_aircraft_ is the vehicle's center‐of‐mass acceleration, g the gravitational field, and a_non‐ideal_ aggregates small contributions from aerodynamic drag, control inputs, vibrations, and cabin disturbances. When the aircraft follows the ballistic segment of the parabola so that a_aircraft ≈ g and the disturbance term satisfy |a_non‐ideal| ≪ |g| (typically ≲ 10^−^^2^ g in parabolic flight), the specific force satisfies |f| ≪ |g| and therefore f ≈ 0; the payload is effectively weightless even though gravity itself is not zero. This condition is referred to as microgravity (micro‐g).

Figure 1b shows images of the experimental setup used in our parabolic‐flight campaigns. All laser–aerogel interactions were conducted inside a custom DN100 CF (100 mm I.D., 250 mm length) stainless‐steel vacuum chamber housed in an aluminum flight enclosure mounted on the interior frame of the aircraft and evacuated with a turbomolecular/diaphragm pump stack to pressures below ∼10^−^^4^ mbar (∼10^−^^2^ Pa) during each parabola. A continuous‐wave 532 nm, 5 W DPSS laser was mounted beneath the chamber on a motorized linear stage so that the beam could be stepped between five fixed borosilicate glass tubes, allowing controlled illumination of each sample during the ∼20 s microgravity window. Graphene aerogel coupons (10 × 10 × 5 mm^3^) were loaded into tapered borosilicate tubes (14.5→9 mm I.D.) and then inverted onto a flat glass plate, which fully confined the samples and prevented ejecta from reaching the pump line during both micro‐g and hyper‐g phases. To minimize external disturbances, the enclosure was installed in a low‐disturbance zone of the cabin, and experimental timing was synchronized with the flight crew to coincide with the microgravity segment of each parabola; prior to every parabola, the samples were reset in the mechanism to avoid premature displacement. Aerogel motion was recorded with a Photron Fastcam Mini UX100 high‐speed camera fitted with a 75 mm f/2.8 macro lens operating at 400 fps (1024 × 768 px, 1/5000 s exposure), illuminated by concentric LED ring lights synchronized to the camera trigger, with additional views used to monitor trajectories and resets between runs. A triaxial accelerometer mounted adjacent to the chamber sampled local g‐levels at 2 kHz to verify the micro‐g window and quantify residual accelerations, while cabin airflow and vibrations were monitored to account for background effects. This setup enabled controlled confinement of the aerogels, repeatable laser delivery from below the samples, and reliable tracking of their motion under weightlessness. Further engineering details and a component‐level schematic of the flight hardware are provided in the Supplementary Information (Figure S6).

### 2.2 Material Characterization

**FIGURE 2:**
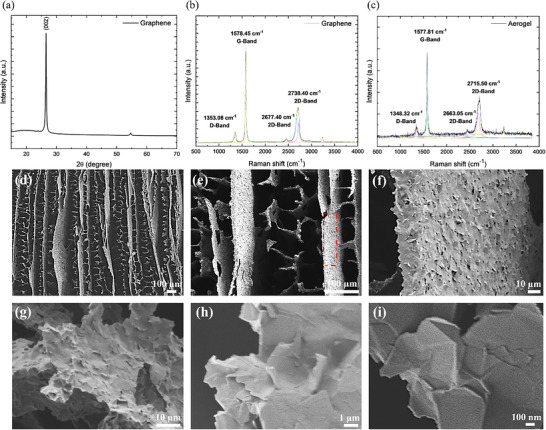
(a) XRD of graphene. (b) Raman of graphene. (c) Raman of graphene aerogels. (d–g) SEM images of AG‐20 graphene aerogels. (h, i) SEM of graphene aerogels at higher magnifications.

Figure 2a shows that the XRD of the graphene sample is dominated by a single broad reflection at 2θ = 25–26°, corresponding to an interlayer spacing of ≈0.340 nm, indexed to the graphitic (002) plane [21], and with no discernible low‐angle (001) peak of graphene oxide near 10 –12° [22]. Raman spectra of graphene samples and the corresponding aerogels fabricated from them are shown in Figure 1b,c. It shows the D, G, and 2D peaks (∼1353 cm^−1^, ∼1578 cm^−^^1^, ∼2738 cm^−1^) in the graphene; and (∼1348 cm^−^^1^, ∼1577 cm^−^^1^, ∼2715 cm^−^^1^) in AGs. The G band arises from the first‐order, in‐plane bond‐stretching optical phonon of E_2_g symmetry at the Brillouin‐zone center [23]. The D band corresponds to the breathing vibration of six‐membered rings with A_1_g symmetry and is activated in Raman only via the intervalley double‐resonance process that couples phonons near K with elastic scattering from defects or edges [24]. The 2D band, also called G′, is the overtone of the D band and originates from a two‐phonon double‐resonance process involving phonons near K [25]. This demonstrates the successful fabrication of graphene aerogel samples and their consistency across different densities (Supplementary Figure S1). Figure 2d–f shows SEM images of graphene aerogels. They revea a strongly aligned, hierarchically porous structure of graphene aerogels. In Figure 2d,e, a parallel, long‐range porous channel runs along a single axis, evidencing a microporous architecture. Figure 2f shows a high‐magnification image of these channels, which consist of flaky graphene structures. Figure 2g–i shows a higher‐magnification of those flakes, which reveals the graphene flaky structure within a scale as low as 100 nm. This confirms the successful fabrication of graphene aerogels and their porous structure (Figures S2–S4).

### 2.3 MicroG vs 1G

**FIGURE 3:**
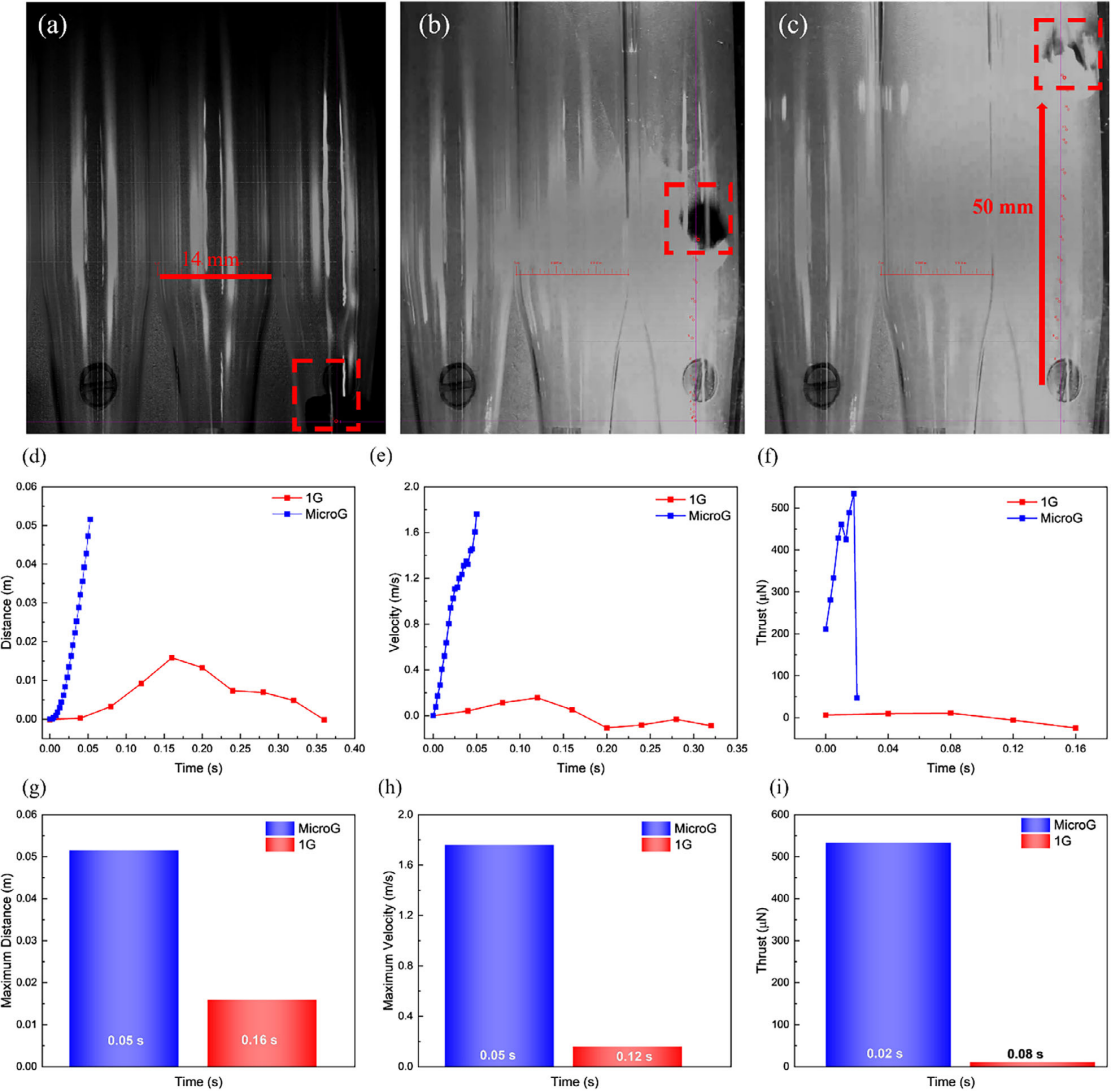
(a) Graphene aerogel with laser off. (b, c) Graphene aerogels move when the laser is on. The comparison between graphene aerogels at 1G vs MicroG in terms of (d) Distance. (e) Velocity (f) Thrust, and the maximum value reached in terms of (g) distance (h) velocity (i) Thrust.

Figure 3 compares the MicroG and 1G conditions. Figure 3a–c shows representative frames with the laser off/on (Figure S5 for the full‐time range). The time traces in Figure 3d (distance) and Figure 3e (velocity) demonstrate that microgravity produces faster and larger responses: displacement grows to ∼0.05 m by ∼0.05 s, and the velocity peaks near 1.7 m s^−^^1^ around the same time. At 1 g, the response is smaller and delayed: displacement peaks near ∼0.015 m at ∼0.16 s, and the velocity reaches only ∼0.06 m s^−^^1^ at ∼0.12 s. The thrust history in Figure 3f follows the same pattern: under microgravity, a strong, early pulse peaks at ≈600 µN (0.6 mN) within ∼0.02–0.03 s and subsequently decays as the aerogels decelerate following impact with the glass tube wall; at 1 g, thrust remains near zero with only a small, delayed maximum (∼11 µN at ∼0.08 s). The bar charts in Figure 3g–i summarize these maxima: distance 0.05 m (microgravity) vs 0.015 m (1 g), velocity 1.7 m s^−^^1^ (microgravity) vs 0.06 m s^−^^1^ (1 g), and thrust 600 µN (microgravity) vs 11 µN (1 g). Overall, microgravity drives the same aerogels to larger displacements and higher velocities, with peak values reached within 0.02–0.05 s compared to 0.08–0.16 s at 1 g, corresponding to a ≈60–75% reduction in the time to reach the maximum response.

## 3 Parametric Study

**FIGURE 4:**
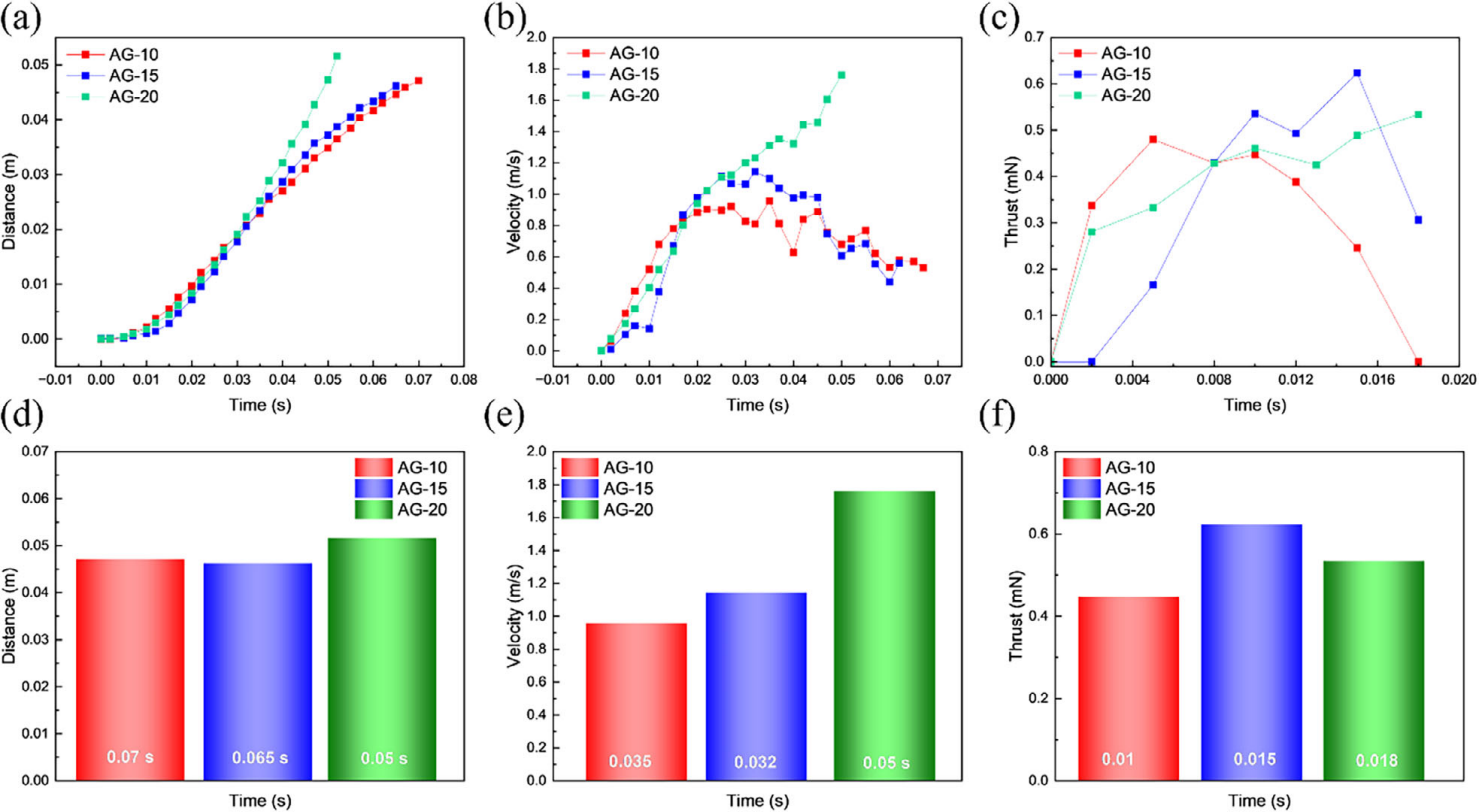
Effect of aerogel density on laser‐induced motion. (a–c) Displacement, velocity, and thrust versus time for AG‐10, AG‐15, and AG‐20. (d–f) Corresponding maxima of displacement, velocity, and thrust, with annotated values indicating time to reach each peak.

Figure 4 summarizes the parametric study for different samples. The parametric study in this section examines how two key design knobs, bulk density of the aerogel and input laser power, govern the light‐driven motion in microgravity. First, we compare three aerogels (AG 10, AG 15, AG 20) that share nominally identical geometry but differ slightly in density and pore structure (0.0074 g cm^−^^3^, 0.0086 g cm^−^^3^, and 0.0098 g cm^−^^3^, respectively), in order to identify which architecture yields the most efficient conversion of light into thrust. Second, we vary the laser power for each aerogel to determine how strongly the response scales with optical input and whether any saturation or threshold behavior emerges. Together, these tests expose how material properties and driving conditions combine to set the achievable displacement, velocity, and thrust.

### 3.1 Effect of Density

To investigate the effect of density on propulsion, we compare three aerogels fabricated from inks with feed concentrations of 10 g L^−^^1^, 15 g L^−^^1^, and 20 g L^−^^1^ (AG‐10, AG‐15, AG‐20), with measured bulk densities of 0.0074 g cm^−^^3^, 0.0086 g cm^−^^3^, and 0.0098 g cm^−^^3^, respectively. In microgravity, all three samples exhibit a similar qualitative response to a laser pulse: displacement grows monotonically over the first ≈0.05–0.1 s, velocity rises to a single peak and then decays, and thrust shows an early pulse followed by weaker oscillations (Figure 4a–c). Quantitatively, AG‐20 achieves the largest and earliest displacements and velocities, whereas AG‐10 consistently shows the smallest response. The thrust histories reveal a more nuanced behavior: AG‐20 displays a relatively smooth rise toward ≈0.6 mN, while AG‐15 produces a sharper, higher peak thrust; this elevated peak is associated with transient acceleration when the sample contacts the glass tube, after which the thrust decays. AG‐10 yields the smallest thrust and decelerates sooner than the others, and the bar charts in Figure 4d–f summarize these trends: AG‐20 leads in distance and velocity, whereas AG‐15 exhibits the highest peak thrust. Overall, these results indicate that simply lowering or raising the density does not monotonically increase performance; the propulsion force exhibits a non‑monotonic dependence on aerogel density. SEM analysis reveals pore throat sizes of approximately 0.39 µm for AG‐15 and ∼45 nm for AG‐20. Extremely low‑density aerogels exhibit very high porosity but reduced structural connectivity, while higher‑density aerogels exhibit smaller pores and increased thermal conductivity. The intermediate‑density aerogel therefore provides an optimal balance between pore connectivity and the formation of transient temperature gradients following laser irradiation. The hierarchical pore architecture enables efficient gas–surface interaction while maintaining transient temperature gradients across the aerogel network, enhancing rarefied gas–surface momentum transfer processes. This picture is consistent with the thermal–gas‑dynamic model (Section S1): in AG‐15, the combination of pore size and porosity yields efficient Knudsen pumping and photophoretic forces, whereas in AG‐10 the lower mass but weaker gas coupling leads to smaller forces, and in AG‐20 the much smaller pores push the system to very high Knudsen number where thermal creep and photophoretic efficiency start to decrease. Thus, the parametric density sweep suggests that there is an optimal intermediate architecture (here represented by AG‐15) for maximizing light‑driven thrust.

### 3.2 Effect of Laser Power

**FIGURE 5:**
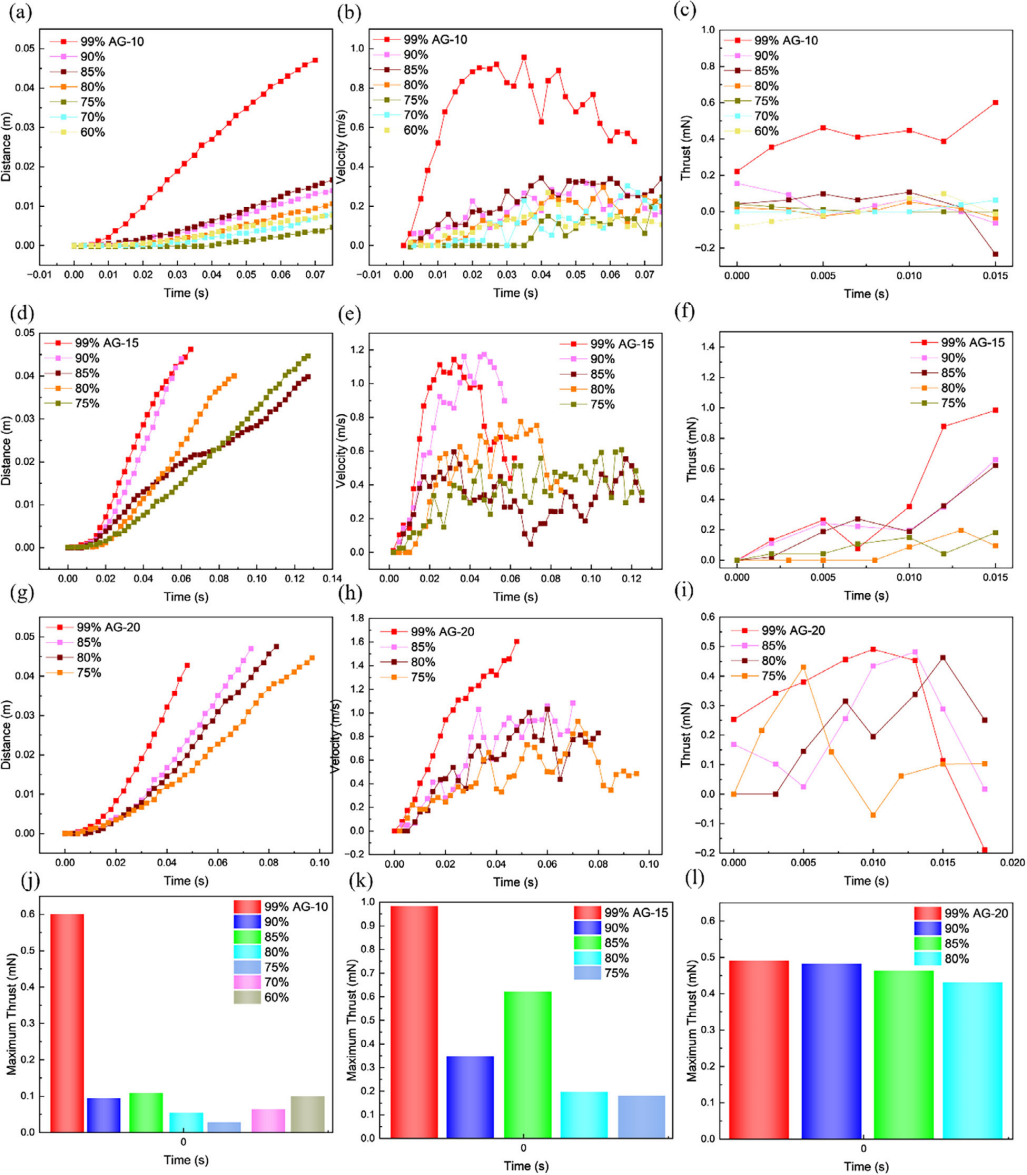
Effect of laser power on the graphene aerogels. (a) Distance (b) Velocity (c) Thrust for AG‐10. (d) Distance (e) Velocity (f) Thrust for AG‐15. (g) Distance (h) Velocity (i) Thrust for AG‐20. Maximum Thrust for (j) AG‐10 (k) AG‐15 (l) AG‐20.

Panels (a–c) in Figure 5 show AG‐10, (d–f) AG‐15, and (g–i) AG‐20; bar charts in (j–l) summarize the maxima. For AG‐10, distance increases strongly with power: at the highest power level, corresponding to 99% of the maximum laser output, the displacement is significantly larger than for the 90–60% power levels (Figure 5a), with velocity and thrust following the same trend (Figure 5b,c). For AG‐15, the 99% and 90% cases produce comparable distances, both exceeding 85–75% (Figure 5d); velocity mirrors this behavior (Figure 5e), while thrust is highest at 99% (Figure 5f). For AG‐20, the distance at 99% is the largest (Figure 5g); the 90% run experienced an in‐flight acquisition gap and fell outside the high‐speed camera's capture window, so that the trace is incomplete. Velocity follows the same trend (Figure 5h). Thrust shows limited sensitivity to power for AG‐20 (Figure 5i): 99% attains the highest value, but the differences across powers are small. The bar charts in Figure 5j–l compare maxima across powers and aerogels: AG‐15 achieves the highest peak thrust overall, followed by AG‐10 and AG‐20.

Taken together, the power sweeps demonstrate that optically driven motion in microgravity is strongly tunable via the incident laser power. For all three aerogels, increasing the power from 60–70 % to ≈99 % yields substantially larger displacements, higher peak velocities, and stronger thrust pulses, with no clear indication of saturation at the highest accessible power. The individual traces also exhibit some shot‐to‐shot variability and occasional spikes at intermediate powers (particularly for AG‐20), indicating sensitivity to sample orientation and image‐tracking window alignment. Nevertheless, the relative ranking of the aerogels is preserved across all powers: AG‐15 consistently produces the largest peak thrust, whereas AG‐20 generally attains the greatest displacement and velocity. From a design perspective, these observations suggest that optical power can be used as an effective control parameter for tuning performance, while the selected aerogel architecture (e.g., AG‐15‐like versus AG‐20‐like) ultimately determines the achievable thrust‐to‐mass ratio.

### 3.3 Effect on the Acceleration

**FIGURE 6:**
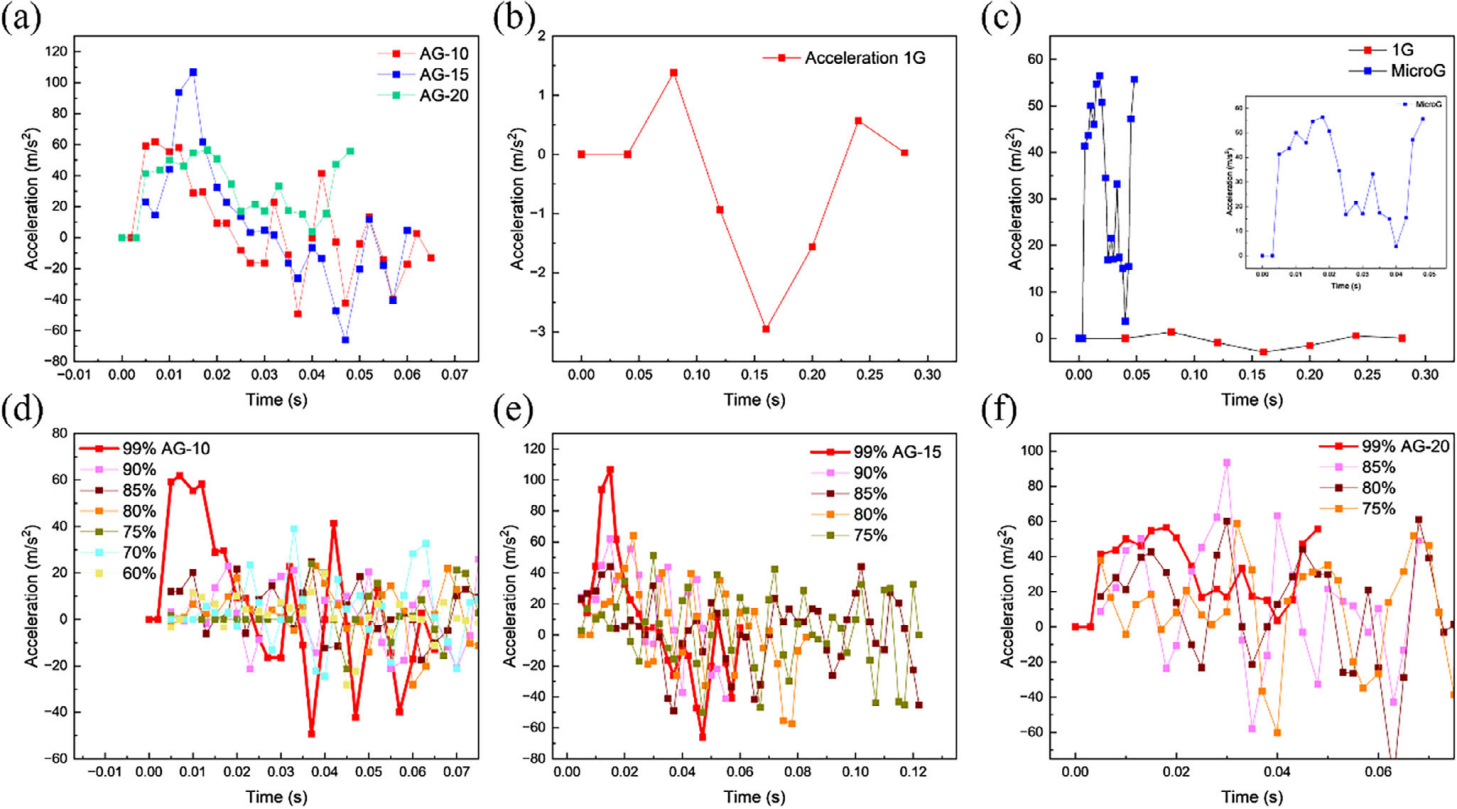
Acceleration response of graphene aerogels under microgravity and 1 g. (a) Microgravity acceleration histories for AG 10, AG 15, and AG 20 at 99% laser power. (b) Corresponding 1 g acceleration history for AG 15. (c) Overlay of AG 15 acceleration at microgravity and 1 g for direct comparison. (d–f) Dependence of the microgravity acceleration on laser power for AG 10, AG 15, and AG 20, respectively.

Figure 6 summarizes the acceleration response. In microgravity (Figure 6a), the laser pulse generates a sharp early‐time positive acceleration peak within ≈10–30 ms. AG‐15 reaches the largest peaks (≈10^2^ m s^−^^2^), whereas AG‐10 and AG‐20 peak at more moderate values of ≈40–60 m s^−^^2^. After this peak, the acceleration decreases, crosses zero, and becomes negative as the aerogels slow down and begin to decelerate against the tube wall. Under 1 g (Figure 6b), the AG‐15 acceleration remains small (|a| ≲ 2 m s^−^^2^) and varies only slowly in time. Figure 6c highlights this contrast by overlaying the AG‐15 traces: microgravity produces accelerations of tens to >100 m s^−^^2^, whereas the corresponding 1 g values stay close to zero.

For AG‐10, Figure 6d shows that the acceleration scales strongly with laser power: the 99 % case shows the largest sustained positive acceleration (≈40–60 m s^−^^2^ in the first 20 ms) and the largest late braking pulses (down to roughly −60 to −80 m s^−^^2^), while traces at 90–60 % remain within ±20–30 m s^−^^2^. For AG‐15 (Figure 6e), the scaling is even more pronounced: 99 % yields the highest peaks (>100 m s^−^^2^) within ≈0.02 s; 90–85 % are intermediate (∼30–60 m s^−^^2^), and 80–70 % are weaker and more oscillatory. For AG‐20 (Figure 6f), the 99 % case provides the highest sustained acceleration (∼20–60 m s^−^^2^), but intermittent spikes appear at intermediate powers (e.g., 85 %), suggesting run‐to‐run variability (e.g., orientation or tracking‐window effects) and an overall weaker power sensitivity than AG‐10/15.

Acceleration in microgravity is therefore dramatically amplified relative to 1 g and generally increases with laser power, with AG‐15 the most responsive. These trends consolidate the distance/velocity/thrust results and reinforce that removing weight and normal‐force friction unlocks much larger optically driven body forces in the aerogels.

To convert these acceleration histories into thrust, we use the relation.

$$F_{\mathrm{th}}\left(t\right)=\;m\;a\left(t\right)$$

where m is the measured mass of each aerogel coupon and a(t) is its instantaneous acceleration in microgravity. For every run, we compute F_th_(t)from the micro‐g acceleration trace and define the “peak thrust” as the maximum value attained in the first ≈10–30 ms after the laser pulse. We restrict the analysis to this early‐time window because, once the aerogel begins to interact with the tube walls, impacts and friction perturb the motion and would introduce systematic errors into the thrust estimate. This procedure is equivalent to the formulation summarized in Equation S1.

## 4 Proposed Mechanism for Light‐Driven Propulsion of Graphene Aerogels

Since the original article of macroscopic “light‐propulsion” in graphene sponges (centimeter, milligram scale) by Zhang et al. in 2015 [16], three thrust mechanisms have been debated: (i) photon radiation pressure, (ii) recoil from light‐induced electron emission, and (iii) gas‐mediated thermal forces (photophoretic/Knudsen effects). This is followed by the work of Wu et al. [17], which showed that the electron‐recoil force inferred from the reported photocurrents (∼9 × 10^−^^7^ A) and electron energies (∼70 eV) is only ∼2.7 × 10^−^^1^ ^1^ N—five or more orders of magnitude below what would be needed to move mg‐scale sponges. That critique redirected attention to radiometric/photophoretic physics in rarefied gas.

Quantitative metrology followed. Wang et al. [18] measured µN‐class forces on graphene sponges with a pendulum apparatus and found their pressure and power dependences match Knudsen‐force predictions (thermal creep through pores under a light‐induced temperature gradient). A companion study [26] reported that the effect is significant at a few pascals (“rough” vacuum) but collapses in high vacuum (∼2 × 10^−^^3^ Pa)—behavior that refutes an electron‐recoil mechanism (which should be largely pressure‐independent) and supports Knudsen/photophoretic propulsion.

Finally, “light‐sail” micro‐membranes made from graphene on a perforated grid were accelerated with 450/655 nm lasers at 0.1–1 W under vacuum and in microgravity [20], showing that atomically thin carbon can be pushed by light at ultralow areal mass. These tests emphasize photon pressure for space‐like conditions, but—crucially for lab/atmospheric environments—also illustrate how thermal gradients can add forces beyond ideal photon pressure if not eliminated.

Under illumination, the strongly absorbing front face of the aerogel heats rapidly while the interior and back face remain cooler for tens of milliseconds because of the material's low effective thermal conductivity and hierarchical porosity. This through‐thickness temperature gradient drives (i) through‐pore thermal transpiration (Knudsen Force). Molecules near the hotter regions gain higher kinetic energy and exert greater momentum, and (ii) external photophoretic/radiometric forces on the aerogel's outer surface due to asymmetric surface momentum. The two contributions add to produce a net thrust along the beam direction. In microgravity, removal of weight and normal‐force friction allows this gas‐mediated thrust to accelerate the samples freely, which is consistent with the observed early mN‐class pulse (∼0.6 mN within ∼0.02–0.03 s) and peak velocities ∼1.7 m s^−^^1^ over ∼0.05 s; in 1 g, the same mechanism is present but largely masked by frictional and buoyancy effects, yielding only ∼11 µN peak thrust at ∼0.08 s and ∼0.06 m s^−^^1^ peak velocity.

A simple energy‐balance model clarifies the origin of this temperature gradient (Section S1). For a single 8 ms, 5‐W laser pulse, the absorbed optical energy is Q_abs_ = P_laser_Δt = 0.04 J. Using an effective specific heat of ≈700 J kg^−^^1^ K^−^^1^ and the measured aerogel masses, this energy raises the temperature of only a thin layer near the illuminated face by several hundred kelvin, while the back face remains close to room temperature. The corresponding thermal diffusion length during the pulse is $\ell_{\mathrm{diff}}-{\left(4\alpha \Delta t\right)}^{\frac{1}{2}}=0.1-0.3$ mm; only ≈2–6 % of the 5 mm thickness, so the heating is strongly localized. As a result, a large through‐thickness temperature difference ΔT is established across the porous network on the timescale relevant for the observed acceleration pulse.

$$F_{net}=Knudsen\;force+Photophoretic\;force$$

$$F_{net}=\Delta_{\mathrm{pKT}}\left(P_{abs},\;\Delta T,K_{n},\alpha \right)A_{\mathrm{eff}}+F_{ph}\left(P_{abs},\Delta T,K_{n},\alpha \right)$$

In this formulation, Δ_pKT_ denotes the thermally driven pressure rise resulting from the Knudsen force through the aerogel's interconnected micropores. When a temperature gradient (∆T) is established between the illuminated and shadowed surfaces, it generates a nonuniform distribution of molecular energy. Molecules near the hotter regions gain higher kinetic energy and exert greater momentum upon the pore walls, while cooler regions sustain lower molecular activity. This imbalance produces a net pressure differential between the two sides of the pore network. The magnitude of this pressure rise depends on the absorbed optical power (P_abs_), the energy‐accommodation coefficient (α), which characterizes the degree of thermal equilibrium between molecules and the solid surface, and the Knudsen number, *K_n_* =  λ/L_throat,_ defined as the ratio of the gas mean free path (λ) to the characteristic pore throat dimension (L_throat_).

SEM analysis reveals that the graphene aerogels possess pore throat sizes spanning from tens of nanometers to sub‐micrometer scales. Under the experimental pressure conditions (10^−^^6^–10^−^^4^ torr), the mean free path of gas molecules is several orders of magnitude larger than the characteristic pore dimensions. This yields Kn ≫ 1, confirming that the system operates in the free‐molecular regime where gas–surface interactions dominate.

The term A_eff_ represents the effective illuminated area of the aerogel that experiences the resultant pressure difference. The products Δ_pKT_ and A_eff_ thus yield the net through‐pore thrust associated with Knudsen pumping. This contribution scales with the absorbed optical power, rather than the incident power, since only absorbed photons contribute to the formation of a temperature gradient.

The second term, F_ph_, accounts for external photophoretic forces acting on the aerogel's outer surfaces due to asymmetric surface momentum exchange in the presence of the same temperature gradient. Together, these two effects form a coupled photothermal–dynamic movement mechanism, wherein absorbed light energy is converted into directed flow and net mechanical thrust. The full derivation of the thermal‐gas‐dynamic model, including the thermal analysis, Knudsen‐number estimates, force calculations, and verifications, is provided in the Supporting Information.

The present parabolic flight experiments were conducted at a nearly fixed‐ chamber pressure of order 10^−4^ torr(∼ 0.02 Pa), so the parametric study focuses on aerogel density and laser power at essentially constant rarefaction level. The role of ambient pressure has been examined separately in our previous ground‐based investigation [27], where the vacuum level was systematically varied while maintaining‐ the aerogel architecture and laser configuration. In that study, the displacement efficiency and measured thrust were found to increase monotonically with increasing vacuum level (decreasing pressure), culminating in a maximum thrust of 36 µN at 10^−5^ torrunder a 3.5 W laser. These results, together with the current microgravity measurements, indicate that the gas‐mediated propulsion of graphene aerogels benefits from operating in a deeper vacuum, reinforcing the relevance of this mechanism for future space‐environment propulsion concepts.

## 5 Conclusion

This work systematically examined the optically induced motion of graphene aerogels in a parabolic‐flight microgravity environment and benchmarked it against 1 g ground behavior. Microgravity produced markedly larger and faster responses: ∼50 mm displacement by ∼0.05 s and peak velocities ≈1.7 m s^−^^1^, accompanied by an early thrust pulse ≈0.6 mN. Under 1 g, the same samples reached only ∼15 mm by ∼0.16 s, with a peak velocity ≈0.06 m s^−^^1^ and a much smaller thrust ≈11 µN at later times. The strong microgravity enhancement of displacement, velocity, and thrust is consistent with a propulsion mechanism arising from a combination of Knudsen pumping and photophoretic forces within the porous graphene network. Collectively, these findings highlight the importance of testing ultralight porous materials in environments that approximate space conditions and indicate that the resulting insights are highly relevant for the development of future space technologies such as laser‐driven micro‐propulsion systems, attitude‐control actuators for small satellites, and ultralight graphene‐based solar sails for photon‐driven spacecraft.

## 6 Materials, Measurements, and Characterizations

### 6.1 Chemicals and Reagents

Graphite flakes (average size of 60–90 µm), perchloric acid (HClO_4_, 70 wt.%), deionized water, and Hydroxy‐ethyl cellulose (HEC, (C_29_H_52_O_21_), 736.71 g/mol).

### 6.2 Synthesis of Expanded Graphite

Expanded graphite was synthesized by adding 2.5 mL of perchloric acid to 1 g of graphite flakes, followed by heating the mixture at 200°C for 30 min to produce a graphite intercalation compound (GIC). The resulting GIC was subsequently exposed to microwave irradiation for 30 s, yielding Thermally Expanded Graphite (TEG).

### 6.3 Synthesis of Graphene Aerogel

Graphene aerogel was synthesized from expanded graphite through a high‐shear exfoliation and freeze‐drying process. Initially, expanded graphite was dispersed in deionized water and HEC and then subjected to exfoliation using a high‐pressure homogenizer operating at a pressure of 500 bar for 50 cycles, forming a stable aqueous graphene dispersion. Three dispersions were made through the same process and denoted as 10g/L, 15g/L and 20g/L. The resulting dispersions were then rapidly frozen by immersion in liquid nitrogen in a silicon mold having the same desired cubic structure to preserve the 3D structure. Finally, the frozen aerogel was subjected to freeze‐drying under vacuum, which sublimated the ice and left behind an ultralight, porous graphene aerogel with a network of interconnected nanosheets.

### 6.4 Characterization and Measurements

Sample morphology was investigated using a Nova NanoSEM 650 scanning electron microscope, offering a beam resolution of 0.8 nm. They were also characterized using a Phoenix Nanotom M high‐resolution micro‐CT system, which is equipped with a 180 kV/20 W nanofocus X‐ray tube and a high‐resolution DXR detector, enabling non‐destructive 3D imaging at voxel resolutions down to 200 nm. Raman spectroscopic measurements on graphene aerogel samples and inks were performed using a Renishaw spectrometer, utilizing a 532 nm excitation laser (2.33 eV) and a 50× objective lens. X‐ray diffraction (XRD) analysis was carried out using an MSAL‐XD2 diffractometer with Cu Kα radiation, while X‐ray photoelectron spectroscopy (XPS) data were acquired with a Thermo Scientific K‐Alpha instrument. The freeze‐drying step was performed using a vacuum freeze‐dryer maintained at 2 Pa and –80°C.

## Conflicts of Interest

The authors declare no conflicts of interest.

## Supporting information




**Supporting File 1**: advs75050‐sup‐0001‐SuppMat.docx.

**Supporting File 2**: advs75050‐sup‐0002‐VideoS1‐S3.zip.

## Data Availability

The data that support the findings of this study are available on request from the corresponding author. The data are not publicly available due to privacy or ethical restrictions.
