# Supplementary material for: Light‑Driven Propulsion of Graphene Aerogels in Microgravity
Source: Adv Sci (Weinh). 2026 Mar 31;13(33):e75050. doi: 10.1002/advs.75050 (PMC13271641; doi:10.1002/advs.75050)
Supplement: Supplementary file 1 — Supporting File 1: advs75050‐sup‐0001‐SuppMat.docx. [file ADVS-13-e75050-s001.docx]

**Light‑Driven Propulsion of Graphene Aerogels in Microgravity**

O. Khattab^a#^, R. Elkaffas^a#^, B. Altawil^a^, O. Alsuwaidi^a^, A. Almubashir^a,b^, Claire Perfetti^c^, S. Shajahan^a^, M.Braibanti^d^, S. Swei^a^, C.S. Iorio^c^, Y. Abdul Samad^a,e,f*^

‘??:

^a^Department of Aerospace Engineering, Khalifa University of Science and Technology, Abu Dhabi, 127788, United Arab Emirates.

^b^ Khalifa University Space Technology and Innovation Lab, Khalifa University, Abu Dhabi, United Arab Emirates.

^c^Centre of Research Engineering and Space Technology, Université libre de Bruxelles, 1050 Brussels, Belgium

^d^European Space Agency (ESA), ESTEC, Noordwijk, the Netherlands

^e^Cambridge Graphene Center, University of Cambridge, Cambridge, CB3 0FA UK.

^f^Advanced Research and Innovation Center (ARIC), Khalifa University, Abu Dhabi, United Arab Emirates.

*Corresponding author: [yy418@cam.ac.uk](mailto:yy418@cam.ac.uk) [Yarjan.abdulsamad@ku.ac.ae](mailto:Yarjan.abdulsamad@ku.ac.ae)

^#^ Authors contributed equally to this work.


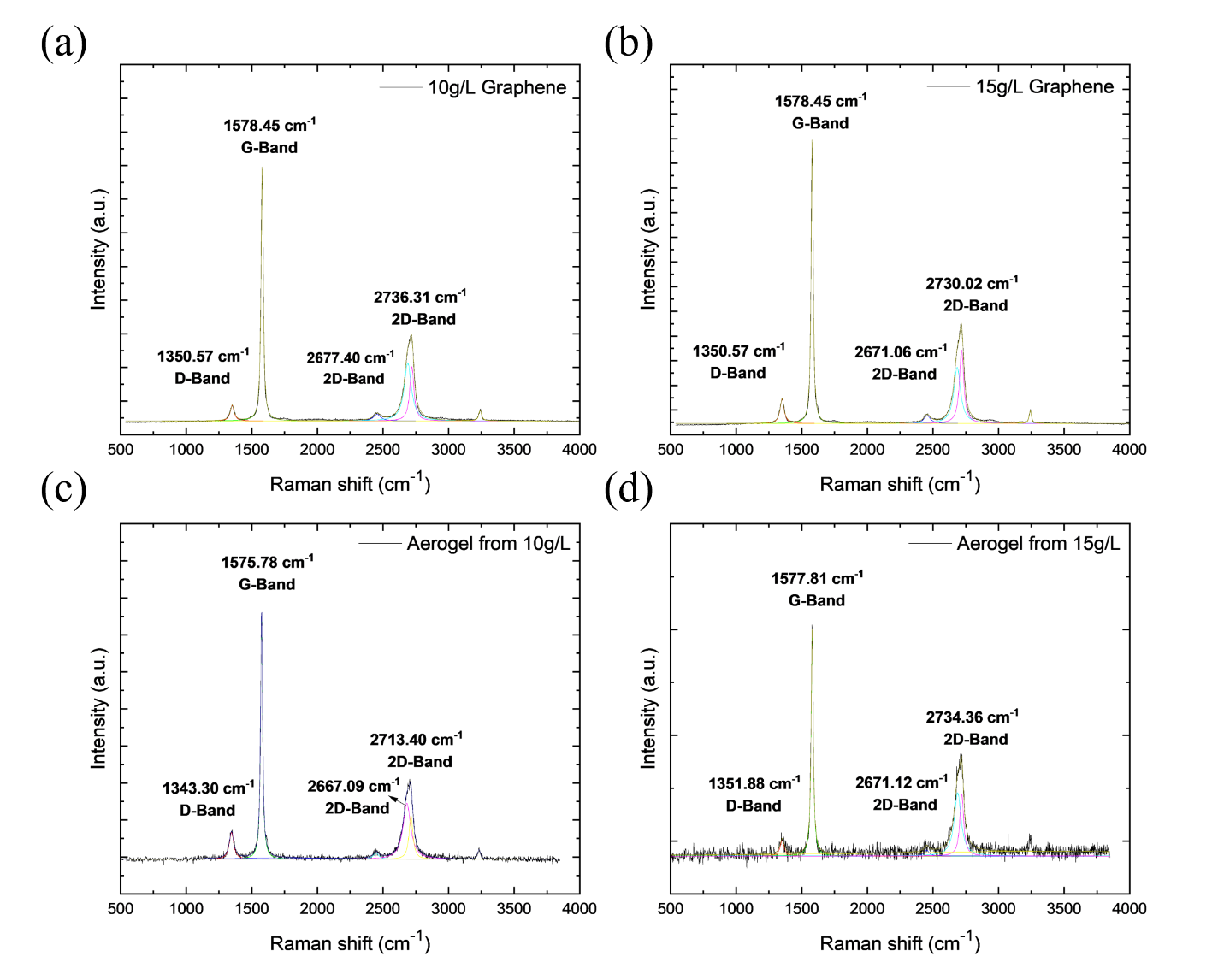


Figure S1 (a,b) Raman of graphene made from 15g/L ink (c,d) and their corresponding aerogels.


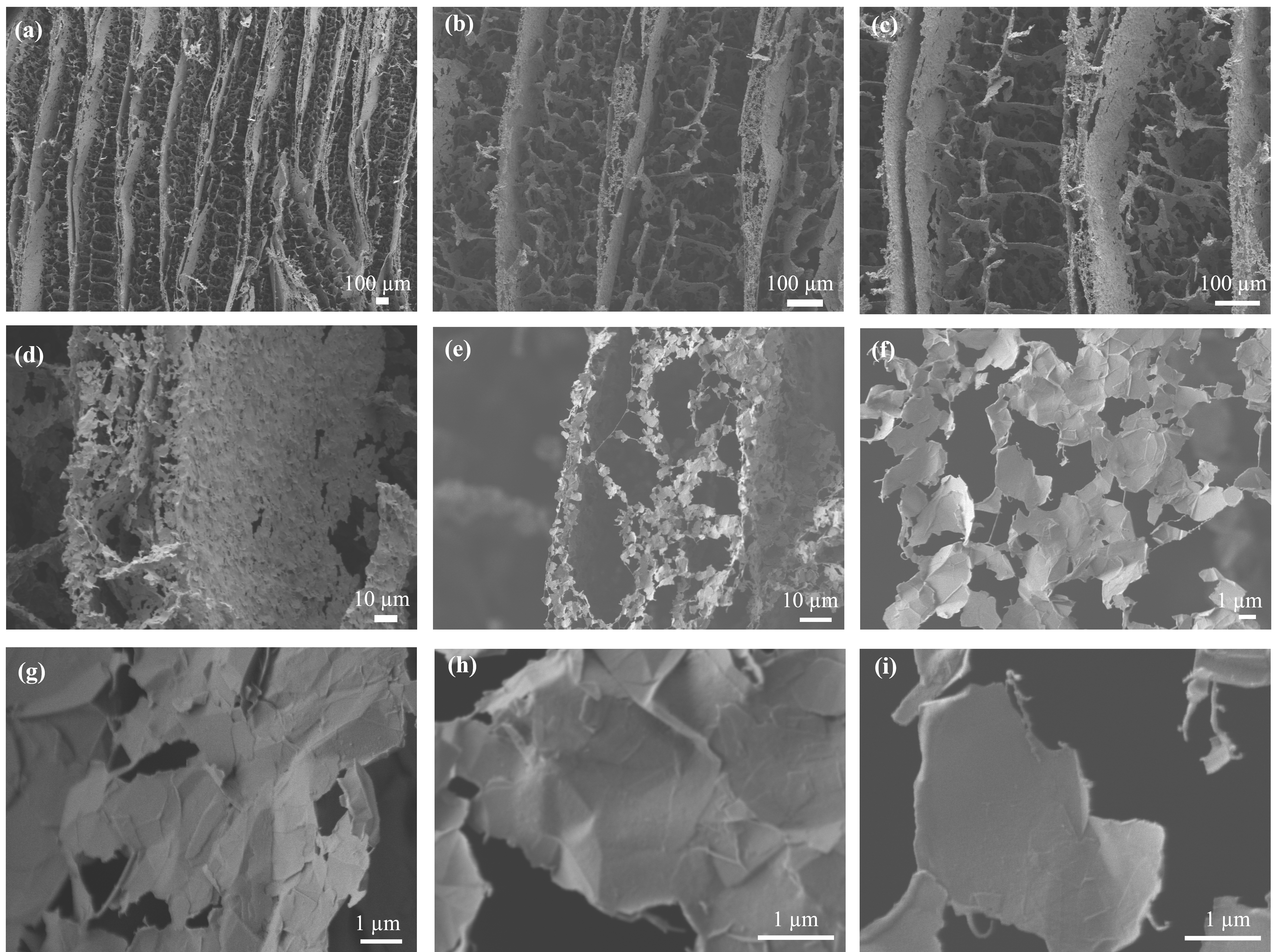


Figure S2 SEM of AG-10.


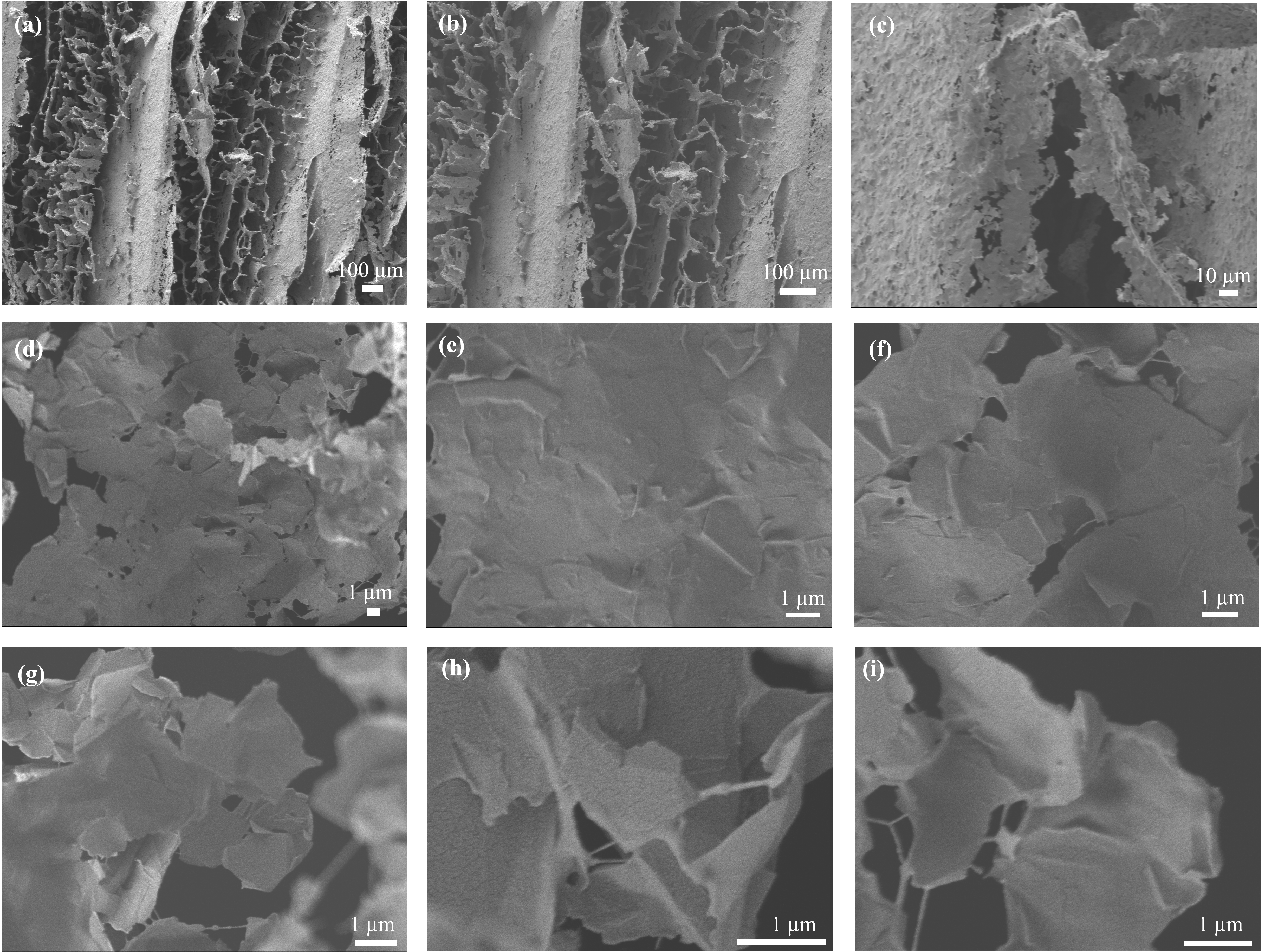


Figure S3 SEM of graphene AG-15.


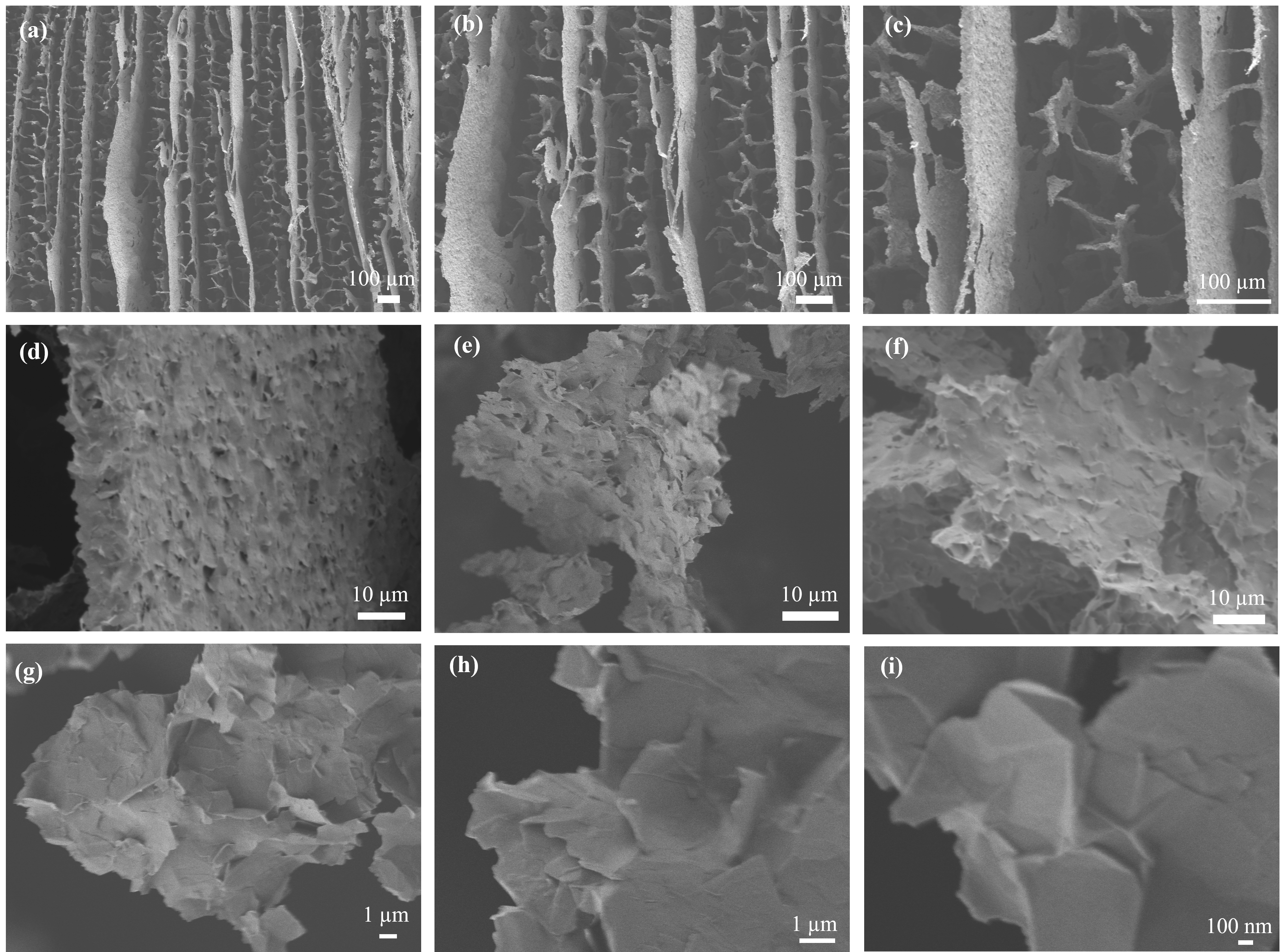


Figure S4 SEM of AG-20.


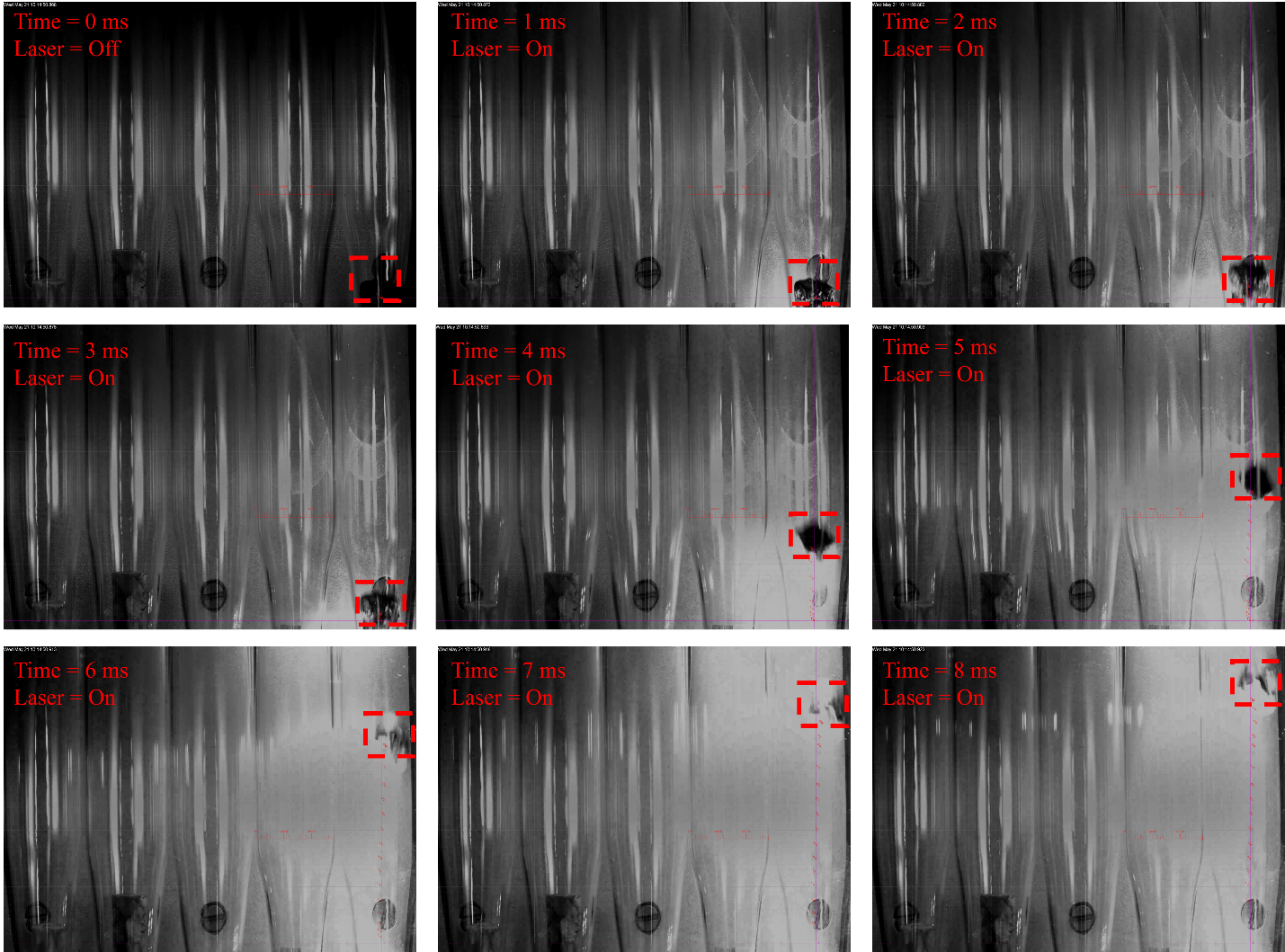


Figure S5 Sample moving inside the micro-G and the calculation of distance.

**
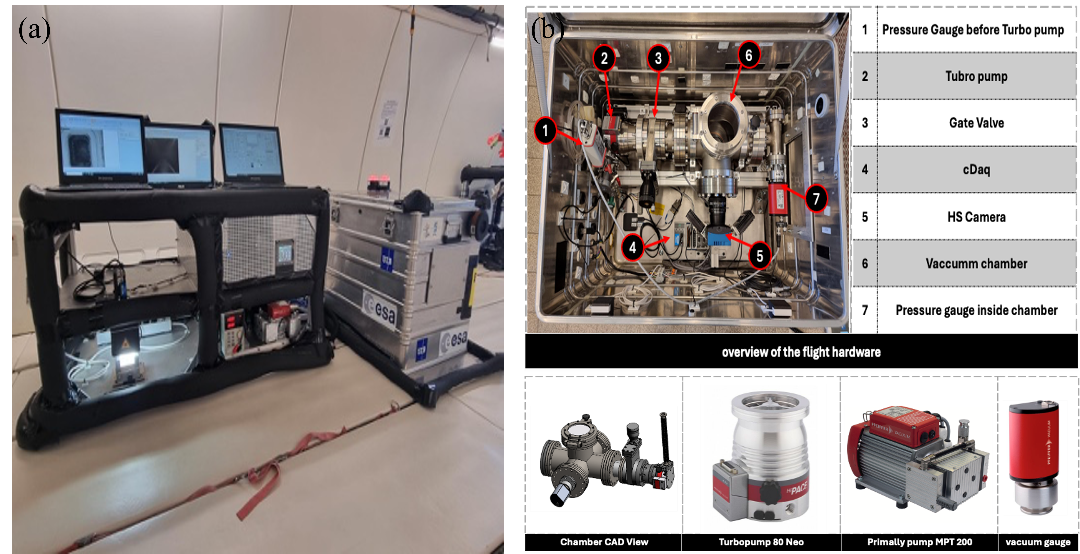
**

Figure S6 (a) Digital Images of the experimental setup on the plane. (b) A detailed component description of the setup.

**Parabolic Flight Mechanism Calculation**

1. **Experimental conditions and laser parameters**

Room temperature:

Chamber pressure: Laser:

T_0_ = 25◦C = 298 K

P = 10−2 Pa

- - DPSS laser wavelength: *λ*_laser_ = 532 nm
  - Nominal laser power: *P*_laser_ = 5 W
  - Pulse duration: ∆*t* = 8 ms = 0*.*008 s

Illuminated front-face area (effective area), approximated from volume:

*A*eff = 10*−*4 m2 We consider a single pulse in the observation window.

1. **Aerogel samples: geometry and masses**

Sample volume:

Densities:

*V* = 10 × 10 × 5 = 0*.*01 × 0*.*01 × 0*.*005 m3 = 5 × 10*−*7 m3

AG-10: *ρ*_10_ = 0*.*0074 g/cm^3^ = 7*.*4 kg/m^3^

AG-15: *ρ*_15_ = 0*.*0086 g/cm^3^ = 8*.*6 kg/m^3^

AG-20: *ρ*_20_ = 0*.*0098 g/cm^3^ = 9*.*8 kg/m^3^

Masses (*m* = *ρV* ):

*m*_10_ = *ρ*_10_*V* = 3*.*7 × 10*−*6 kg*,*

*m*_15_ = *ρ*_15_*V* = 4*.*3 × 10*−*6 kg*,*

*m*_20_ = *ρ*_20_*V* = 4*.*9 × 10*−*6 kg*.*

1. **Thermal and force model: general assumptions**

We assume for the thermal and force model:

laser power ⇒ temperature rise ⇒ Knudsen pumping + photophoretic force*.*

1. **Short-time adiabatic heating.** During the 8 ms pulse, radiative and conductive heat losses are small due to vacuum and short duration, so the illuminated region heats approximately adiabatically.
2. **Effective homogeneous heating mass.** The effective mass *m*_eff_ of the aerogel near the illuminated face participates in the temperature rise. For a lower-bound estimate we can take *m*_eff_ = *m_i_* (whole mass), but in reality only one face is heated, so the heating is strongly localised: layers closest to the illuminated face experience the greatest temperature rise, while the back face remains much closer to *T*_0_ during the 8 ms pulse.
3. **Effective heat capacity.** The aerogel’s effective specific heat is approximated by a graphite-like value [1]

*C*_eff_ ≈ 700 J kg*−*1K*−*1*.*

1. **Absorbed vs. incident power.** A fraction *η* of the laser power is absorbed:

*P*_abs_ = *η P*_laser_*,* 0 ≤ *η* ≤ 1*.*

Based on graphene aerogel features (extremely black, highly porous, strongly absorptive at 532 nm, low reflectivity and low transmissivity), and from SEM, Raman and XRD measurements, graphene absorbs more than 90% of visible light. Graphene-based aerogels absorb ∼ 75% of green laser light. Thus almost all laser energy entering the tube is absorbed, and for an upper bound on ∆*T* we take *η* ≈ 1.

1. **Force scaling in our pressure regime.** At *P* ∼ 10*−*2 Pa, both through-pore Knudsen pumping and external photophoretic force scale approximately linearly with the normalized temperature rise ∆*T/T*_0_ for modest ∆*T* .
2. **Clean force extraction in microgravity.** In microgravity (*µg*), weight and normal- force friction are strongly suppressed, so the initial acceleration reflects predominantly the optically induced gas-mediated thrust.
3. **Temperature rise: energy balance and effective thickness**
4. **Basic energy balance.** Over a single laser pulse of duration ∆*t*, the absorbed optical energy is

*Q*abs = *P*abs∆*t* = *ηP*laser∆*t.*

Assuming this energy raises the temperature of an effective mass from *T*_0_ to *T_s_* = *T*_0_ + ∆*T* :

*Q*abs = *m*eff*C*eff∆*T,*

so

∆*T* = *ηP*laser∆*t .*

*m*eff*C*eff

1. **One-face heating and effective fraction** *f* **.** For one-face heating we take

*m*_eff_ = *fm_i_,* 0 *< f* ≤ 1*,*

where *f* = 1 corresponds to uniform heating of the entire sample, and *f <* 1 to heating of only a fraction of the thickness. Then

$$T_{i}=\frac{\eta P_{laser}\Delta t}{fm_{i}C_{eff}}$$

1. **Thermal diffusion length.** The thermal diffusion length during the 8 ms pulse is [2]

$$\mathcal{l}_{\mathrm{diff}}= \sqrt{\alpha\Delta t}$$

where *α* is the thermal diffusivity of the aerogel.

Using an effective thermal conductivity for graphene aerogel *κ* ≈ 0*.*02–0*.*05 W/m K [3], density

*ρ* ≈ 8–10 kg/m^3^, and heat capacity *C* = 700 J kg*−*1K*−*1, we obtain

*α* =  *κ* 10*−*5–10*−*6 m2/s [4]*.*

≈

*ρC*

Thus $\mathcal{l}_{\mathrm{diff}} \approx\sqrt{\alpha\Delta t}\approx\sqrt{\left( {10}^{-5}-{10}^{-6} \right)*8*{10}^{-3}} \approx{10}^{-4}-3*{10}^{-4}m$

The heat therefore penetrates roughly 0*.*1–0*.*3 mm into the aerogel during the laser pulse. Since the sample thickness is 5 mm, the fraction of thickness strongly heated is

*f*_thick_ =  *ℓ*diff ≈

5

0*.*1 – 0*.*3

5 mm

≈ 0*.*02–0*.*06

i.e. about 2–6% of the thickness. We take a representative value *f* ≈ 0*.*04.

1. **Numerical temperature rise.** With *η* = 1, *P*_laser_ = 5 W, ∆*t* = 8 ms, *C*_eff_ = 700 J kg*−*1K*−*1, and *f* = 0*.*04, we estimate for each sample using:

$$T_{i}=\frac{\eta P_{laser}\Delta t}{fm_{i}C_{eff}}$$

AG-10: ∆*T* ≈ 386 K*,*

AG-15: ∆*T* ≈ 332 K*,*

AG-20: ∆*T* ≈ 292 K*.*

The surface temperature is

*T_s_* = *T*_0_ + ∆*T_i_,*

so the front-face temperature rise is in the rough range 600–700 K.

1. **Experimental net force from acceleration curves**

From acceleration–time curves in microgravity (*µg*) at 99% laser power:

AG-10: *a*_0_*_,_*_10_ ≈ 40–60 mm/s^2^ ∼ 50 mm/s^2^*,*

AG-15: *a*_0_*_,_*_15_ ≈ 80–100 mm/s^2^ ∼ 100 mm/s^2^*,*

AG-20: *a*_0_*_,_*_20_ ≈ 20–60 mm/s^2^ ∼ 40 mm/s^2^*.*

The net propulsive force in microgravity is

*F*net*,i* = *mia*0*,i* (N)*,*

giving

*F*net*,*10 = *m*10*a*0*,*10 = 1*.*85 × 10*−*7 N*,*

*F*net*,*15 = *m*15*a*0*,*15 = 4*.*3 × 10*−*7 N*,*

*F*net*,*20 = *m*20*a*0*,*20 = 1*.*96 × 10*−*7 N*.*

Theoretically, the net force can be expressed as

*F*_net_ = ∆*P*_KT_(*P*_abs_*,* ∆*T,* Kn*, α*) *A*_eff_ + *F*_ph_(*P*_abs_*,* ∆*T,* Kn*, α*)*,*

where:

- ∆*P*_KT_ is the Knudsen-pumping-induced pressure rise across the porous network,
- *F*_ph_ is the photophoretic surface force.

Both depend on the temperature difference between the hot front face and the cooler back region [5-9]

1. **Knudsen and photophoretic force model**

We approximate [5], [10], [11]

$$\frac{\Delta P_{K}}{P}\approx C_{T}\left( \Delta\frac{T}{T_{0}} \right)$$

$$F_{K}\approx C_{T}P A_{eff}\left( \Delta\frac{T}{T_{0}} \right)$$

$$\frac{\Delta P_{ph}}{P}\approx C_{ph}\left( \Delta\frac{T}{T_{0}} \right)$$

$$F_{ph}\approx C_{ph}P A_{eff}\left( \Delta\frac{T}{T_{0}} \right)$$

0

where *C_T_* and *C*_ph_ are geometry–gas-dependent coefficients.

Thus

$$F_{net}={(C}_{T}+C_{ph})P A_{eff}\frac{\Delta T}{T_{0}} (Knudsen+photophoretic)$$

Define an effective Knudsen coefficient

$$C_{K,eff}=C_{T}+C_{ph}$$

For each sample

$$F_{net,i}=C_{K,eff}P A_{eff}\frac{\Delta T_{i}}{T_{0}}$$

So that

$$m_{i}a_{o,i}=C_{K,eff}P A_{eff}\frac{\Delta T_{i}}{T_{0}}$$

And therefore

$$C_{K,eff}=\frac{m_{i}a_{o,i}T_{0}}{P A_{eff}\Delta T_{i}}$$

Using the expression for $\Delta T_{i}$

$$\Delta T_{i}= \frac{\eta P_{laser}\Delta t}{f m_{i}C_{eff}}$$

We can also write

$$C_{K,eff}= \frac{F_{net,i}T_{0}}{P A_{eff}\Delta T_{i}}$$

With *T*_0_ = 298 K, *P* = 10*−*2 Pa, *A*_eff_ = 10*−*4 m2, we obtain:

AG-10: *C_K,_*_eff_*_,_*_10_ = 1*.*43 × 102*,*

AG-15: *C_K,_*_eff_*_,_*_15_ = 3*.*86 × 102*,*

AG-20: *C_K,_*_eff_*_,_*_20_ = 2 × 102*.*

1. **Gas rarefaction and Knudsen numbers**

At our low pressure $P=1*{10}^{-2}$ , gas molecules are far apart and the mean free path is extremely large (on the order of meters)

Using kinetic theory

$$\lambda= \frac{K_{B}T}{\sqrt{2} \pi d^{2}P}$$

with air-molecule diameter ∼ 0*.*36–0*.*37 nm gives [13]

*λ* ≈ 0*.*5–0*.*7 m at 0*.*01 Pa*,* 300 K*.*

From SEM, pore-throat diameters:

- AG-15: ∼ 0*.*39 *µ*m,
- AG-20: ∼ 45 nm (smaller pores than AG-15).

The Knudsen number is [14]

$$K_{n}=\lambda/ (Pore size)$$

measuring how far molecules travel before collision, i.e. the ratio of mean free path to channel size.

For AG-15

$$Kn_{15}\sim\frac{0.7}{3.9*{10}^{-7} m} \approx1.8*{10}^{6}$$

For AG-15

$$Kn_{20}\sim\frac{0.7}{4.5*{10}^{-8} m} \approx1.6*{10}^{7}$$

Thus Kn>> 10 for both, indicating free molecular regime; molecules mostly hit walls not each other [15].

1. **Pulse energy and simple** ∆*T* **estimate**

Known laser power:

*P*_laser_ = 5 W*, λ*_laser_ = 532 nm*,* ∆*t* = 8 ms*.*

Total energy delivered per pulse:

*E* = *P*_laser_∆*t* = 5 × 0*.*008 = 0*.*04 J*.*

Let *η* be the fraction of absorbed energy. For graphene aerogel, *η* ≈ 0*.*9, so

*E*_abs_ = *ηE* = 0*.*9 × 0*.*04 = 0*.*036 J

per pulse.

Take the measured sample masses:

*m*_10_ = 3*.*7 × 10*−*6 kg*, m*_15_ = 4*.*3 × 10*−*6 kg*, m*_20_ = 4*.*9 × 10*−*6 kg*,*

and the specific heat for graphene aerogel

*C* = 700 J kg*−*1K*−*1*.*

As a representative value we take the middle mass, *m* ≃ *m*_15_ = 4*.*3 × 10*−*6 kg, so the heat capacity is

Then

*mC* = (4*.*3 × 10*−*6)(700) ≈ 3*.*0 × 10*−*3 J K*−*1*.*


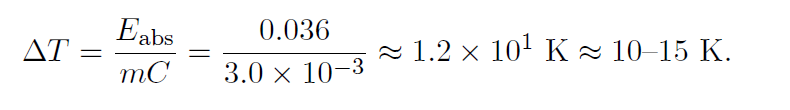


So in this simple uniform-heating estimate, each laser pulse raises the average aerogel temperature by only of order 10 K.

1. **Knudsen and Photophoretic Contributions**

Let χ (Kn) and $\psi\left( \mathrm{Kn} \right)$ denote the Knudsen and photophoretic coefficient, respectively.

Knudsen force:

$$\Delta P_{KT}=P \chi(Kn, \alpha)\frac{\Delta T}{T_{0}}$$

where *χ* is the accommodation coefficient (how well molecules exchange heat with the surface). For Kn ≫ 1 and *α* ≈ 1, we take *χ* ≈ 0*.*5, so

∆*P* 1 ∆*T*

*P* ≈ 2 *T .*

Photophoretic contribution:

$$\Delta P_{\mathrm{Ph}}=P \psi(Kn, \boldsymbol{\alpha)}\frac{\Delta T}{T_{0}}$$

and for a black, highly conducting particle with Kn ≫ 10, we take Ψ ≈ 0*.*5. We have an average projected area

*A* = 7*.*85 × 10*−*5 m2*.*

Resulting force estimates:

AG-15: *F*_KT_ ≈ 2*.*9 × 10*−*7 N*, F*_ph_ ≈ 2*.*2 × 10*−*7 N*,*

AG-20: *F*_KT_ ≈ 1*.*7 × 10*−*7 N*, F*_ph_ ≈ 1*.*7 × 10*−*7 N*.*

Thus

AG-15: *F*_in_ = *F*_KT_ + *F*_ph_ = 4*.*4 × 10*−*7 N*,*

AG-20: *F*_in_ = *F*_KT_ + *F*_ph_ = 3*.*4 × 10*−*7 N

1. **Comparison with experimental forces and discussion**

The comparison

*F*th,15 ≈ *F*exp,15*,*

*F*th,20 *> F*exp,20*,*

shows that the simplified free-molecular model with constant coefficients *χ* ≈ *ψ* ≈ 0*.*5 works for AG–15 but overestimates the force for AG–20.

AG–20 has much smaller pores (∼ 45 nm) and a much larger Knudsen number. Both theory and experiments in related systems indicate that

- - The efficiency of thermal creep (Knudsen pumping) does not increase indefinitely with *K_n_*; at extremely large *K_n_* it can decrease because the gas becomes too rarefied inside the pores;
  - The photophoretic force in the free-molecular limit depends sensitively on the particle’s thermal conductivity and on the exact micro-geometry, and it also tends to decrease at very large *K_n_*.

In other words, for ultra-small pores at very high *K_n_*, the effective coefficients *χ* and *ψ* are smaller than the assumed value 0*.*5. This explains why AG–20 shows a smaller thrust than predicted.

In a low-pressure microgravity environment, short laser pulses strongly heat the front face of porous graphene aerogels. The resulting temperature gradients in a free-molecular gas drive through-pore Knudsen pumping and photophoretic forces, producing measurable thrust of order 10*−*4 N.

For the AG–15 sample, a simple free-molecular model with *χ* ≈ *ψ* ≈ 0*.*5 reproduces the measured force, supporting the interpretation that the motion is dominated by gas-mediated Knudsen and photophoretic effects. For AG–20, which has much smaller pores and a much larger Knudsen number, the measured force is significantly lower than the simple prediction, consistent with a loss of efficiency of thermal creep and photophoretic mechanisms at extremely high *K_n_* and very small pore sizes.

**Experimental set‑up**

**Vacuum module**

All laser–aerogel interactions were performed inside a custom DN100 CF (100 mm I.D.) stainless‑steel chamber (250 mm length) housed in a Zarges K470 aluminum enclosure (Fig. 2). A CF‑flanged stainless‑steel gate valve (Kurt J. Lesker) isolated the chamber from the pump line. High vacuum was provided by a Pfeiffer HiPace 80 Neo turbomolecular pump (PMP 08031110; nominal pumping speed 67 L s⁻¹; DN100 CF inlet) backed by a Pfeiffer MVP 015 diaphragm pump via a DN25 KF roughing line. Chamber pressure was logged continuously with a dual‑range Pfeiffer MPT 200 (PTR 40351) and cross-checked with two capacitance manometers (Pfeiffer CMR 361; 0–1000 mbar and 0–0.1 mbar ranges). After a 30-minute pump-down from ambient, the base pressure reached < 5 × 10⁻⁵ mbar (≈ 5 × 10⁻³ Pa) and remained < 1 × 10⁻⁴ mbar (≈ 1 × 10⁻² Pa) throughout each flight segment; typical pre-flight base was < 5 × 10⁻⁵ mbar. All vacuum components were solvent‑cleaned in a cleanroom and assembled ≥ 24 h before each sortie to minimize outgassing.

**Laser delivery and control**

A continuous‑wave DPSS laser (532 nm, 5 W) was mounted beneath the chamber on a motorized linear stage (travel 0–120 mm; repeatability ± 0.02 mm) to register five programmed positions aligned to five borosilicate glass containment tubes. Laser power gating and timing were controlled via a National Instruments cDAQ‑9174 chassis with a 9402 counter module; triggers were TTL‑referenced to the high-speed camera. The laser was operated in continuous‑wave mode at 100 % setting, corresponding to 5 W output power. The spot diameter at the sample was 0.1 mm (illuminated area $A\approx7.85\times{10}^{-9}\text{ }\text{m}^{2}$), giving an average power density of $6.37\times{10}^{8}\text{ }\text{W m}^{-2}$(63.7 kW cm⁻²). Because the laser is continuous‑wave, the peak power density equals this average value.”

**Aerogel target assembly**

Graphene‑aerogel coupons (10 × 10 × 5 mm³) were loaded into borosilicate tubes with a tapered bore (14.5 → 9 mm I.D.) to reduce residual‑gas flow. After loading, each tube was inverted and seated on a clear borosilicate flat‑glass plate, trapping the coupon against the upper end. This geometry fully confines the sample during laser firing and through micro‑g maneuvers, preventing ejecta from reaching the pump line or internal gauges. To check for possible laser‑induced degradation, each aerogel was weighed before and after the full irradiation sequence; the measured masses were identical within the balance resolution, indicating no detectable material loss (e.g. burning or ablation) under the applied laser conditions.

**Imaging and diagnostics**

Motion was recorded by a Photron Fastcam Mini UX100 fitted with a 75 mm f/2.8 macro lens at 400 fps (1024 × 768 px) with 1/5000 s exposure. Two concentric LED ring lights (24 V; ~50 klx at the working distance) provided uniform illumination and were TTL‑synchronized to the camera. A triaxial accelerometer mounted adjacent to the chamber sampled local g‑levels at 2 kHz through the cDAQ system [model, ±g range, and mounting location to be inserted] to verify the micro‑g window and quantify residual accelerations.

**Control logic and safety interlocks**

A software interlock inhibited laser emission unless pressure < 0.1 mbar and the gate valve open status were both satisfied. A keyed hardware interlock and dual beacon indicators met ESA flight‑safety requirements. To protect the turbopump, power was cut automatically if pressure exceeded 5 × 10⁻⁴ mbar during any inadvertent repressurization.

**Operational timeline (per parabola)**

Before each micro‑g segment, the chamber was pumped from cabin pressure (~800 mbar) to < 1 × 10⁻⁴ mbar in ~8 min. Five seconds before parabola onset (1.8 g entry), the camera and DAQ were armed. High-speed recording began at T = −5 s; a single 8-ms laser pulse was triggered at T ≈ +3 s, near the midpoint of the micro‑g window. Total capture per run was 25s, spanning hyper‑g entry, micro‑g, and pull‑out.

**Ground reference tests**

To contextualize the micro‑g response, matched shots were performed on the ground with the chamber oriented vertically. Aerogel displacement was observed at 1 g under otherwise identical optical conditions, confirming that laser-induced forces dominate over gravitational loading for the tested masses. Quantitative 1 g vs µg comparisons are reported in the Results section.

**References**

[1] E. Pop, V. Varshney, and A. K. Roy, “Thermal properties of graphene: Fundamentals and applications,” *MRS Bulletin*, vol. 37, no. 12, pp. 1273–1281, Dec. 2012, doi: 10.1557/mrs.2012.203.

[2] Carslaw, Horatio Scott. "J. c. Jaeger." *Conduction of heat in solids* 2 (1959).

[3] Y. Xie, S. Xu, Z. Xu, H. Wu, C. Deng, and X. Wang, “Interface-mediated extremely low thermal conductivity of graphene aerogel,” *Carbon*, vol. 98, pp. 381–390, Mar. 2016, doi: 10.1016/j.carbon.2015.11.033.

[4] T. L. Bergman and A. S. Lavine, *Fundamentals of heat and mass transfer*, Eighth edition. Hoboken, NJ: John Wiley & Sons, 2017.

[5] H. Horvath, “Photophoresis – a Forgotten Force ??,” *KONA*, vol. 31, no. 0, pp. 181–199, 2014, doi: 10.14356/kona.2014009.

[6] O. Jovanovic, “Photophoresis—Light induced motion of particles suspended in gas,” *Journal of Quantitative Spectroscopy and Radiative Transfer*, vol. 110, no. 11, pp. 889–901, July 2009, doi: 10.1016/j.jqsrt.2009.02.033.

[7] Han, Y. L., et al. "Knudsen compressor performance at low pressures." *AIP Conference Proceedings*. Vol. 762. No. 1. American Institute of Physics, 2005..

[8] M. Rojas-Cárdenas, I. Graur, P. Perrier, and J. G. Méolans, “Time-dependent experimental analysis of a thermal transpiration rarefied gas flow,” *Physics of Fluids*, vol. 25, no. 7, p. 072001, July 2013, doi: 10.1063/1.4813805.

[9] B. C. Schafer, J. Kim, F. Sharipov, G.-S. Hwang, J. J. Vlassak, and D. W. Keith, “Photophoretic flight of perforated structures in near-space conditions,” *Nature*, vol. 644, no. 8076, pp. 362–369, Aug. 2025, doi: 10.1038/s41586-025-09281-8.

[10] H. Rohatschek, “Semi-empirical model of photophoretic forces for the entire range of pressures,” *Journal of Aerosol Science*, vol. 26, no. 5, pp. 717–734, July 1995, doi: 10.1016/0021-8502(95)00011-Z.

[11] S. Beresnev, V. Chernyak, and G. Fomyagin, “Photophoresis of a spherical particle in a rarefied gas,” *Physics of Fluids A: Fluid Dynamics*, vol. 5, no. 8, pp. 2043–2052, Aug. 1993, doi: 10.1063/1.858540.

[12] “The Molecular Theory of Gases and Liquids - Joseph O. Hirschfelder, Charles F. Curtiss, R. Byron Bird

[13] N. Hedin, L. Chen, and A. Laaksonen, “Sorbents for CO 2 capture from flue gas—aspects from materials and theoretical chemistry,” *Nanoscale*, vol. 2, no. 10, pp. 1819–1841, 2010, doi: 10.1039/C0NR00042F.

[14] “Rarefied Gas Dynamics: From Basic Concepts to Actual Calculations - Carlo Cercignani

[15] X. Wu, Y. Guo, X. Pan, and Z. Yang, “The direct Monte Carlo simulation of microchannel flows for a large Knudsen number range,” *Physics of Fluids*, vol. 36, no. 2, p. 023360, Feb. 2024, doi: 10.1063/5.0193308.
